# Two approaches for estimating the lower limit of quantitation (LLOQ) of microRNA levels assayed as exploratory biomarkers by RT-qPCR

**DOI:** 10.1186/s12896-018-0415-4

**Published:** 2018-02-02

**Authors:** Russell D. Wolfinger, Sudheer Beedanagari, Eric Boitier, Tao Chen, Philippe Couttet, Heidrun Ellinger-Ziegelbauer, Gregory Guillemain, Claire Mariet, Peter Mouritzen, Raegan O’Lone, P. Scott Pine, Tatiana Sharapova, Jian Yan, Peter S. Yuen, Karol L. Thompson

**Affiliations:** 1 SAS Institute Inc., Cary, NC 27513 USA; 2 Alkermes Inc., Waltham, MA 02451 USA; 3 Sanofi R&D, Disposition Safety and Animal Research, Vitry-sur-Seine, France; 4 Division of Genetic and Molecular Toxicology, National Center for Toxicological Research, Food and Drug Administration, Jefferson, AR 72079 USA; 5 Novartis Pharma AG, CH-4057 Basel, CH Switzerland; 6 Toxicology, Bayer Pharma AG, 42096 Wuppertal, Germany; 7 Exiqon, DK-2950 Vedbaek, Denmark; 8 ILSI Health and Environmental Sciences Institute, 1156 15th NW, 2nd Floor, Washington, DC 20005 USA; 9 National Institute of Standards and Technology, Stanford, CA 94305 USA; 10 AbbVie, Abbott Park, IL 60064 USA; 11 NIH/NIDDK, Bethesda, MD 20892 USA; 12 Center for Drug Evaluation and Research, Food and Drug Administration, Silver Spring, MD 20993 USA; 13 Bristol Myers Squibb, New Brunswick, NJ USA

**Keywords:** microRNA, Absolute quantitation, Quantitative PCR, Lower limit of quantitation

## Abstract

**Background:**

Circulating microRNAs are undergoing exploratory use as safety biomarkers in drug development. Reverse transcription quantitative polymerase chain reaction (RT-qPCR) is one common approach used to quantitate levels of microRNAs in samples that includes the use of a standard curve of calibrators fit to a regression model. Guidelines are needed for setting assay quantitation thresholds that are appropriate for this method and to biomarker pre-validation.

**Results:**

In this report, we develop two workflows for determining a lower limit of quantitation (LLOQ) for RT-qPCR assays of microRNAs in exploratory studies. One workflow is based on an error threshold calculated by a logistic model of the calibration curve data. The second workflow is based on a threshold set by the sample blank, which is the no template control for RT-qPCR. The two workflows are used to set lower thresholds of reportable microRNA levels for an example dataset in which miR-208a levels in biofluids are quantitated in a cardiac injury model. LLOQ thresholds set by either workflow are effective in filtering out microRNA values with large uncertainty estimates.

**Conclusions:**

Two workflows for LLOQ determinations are presented in this report that provide methods that are easy to implement in investigational studies of microRNA safety biomarkers and offer choices in levels of conservatism in setting lower limits of acceptable values that facilitate interpretation of results.

**Electronic supplementary material:**

The online version of this article (10.1186/s12896-018-0415-4) contains supplementary material, which is available to authorized users.

## Background

MicroRNAs (miRNAs) that have restricted tissue expression and are released into biofluids upon tissue injury are being evaluated as safety biomarkers potentially diagnostic of site of injury. Tissue-selective miRNAs have been identified by profiling the miRNA content of bodily tissues in humans, mice, and rats [1–3]. Reverse transcription quantitative polymerase chain reaction (RT-qPCR) assays have been adapted for the detection of very low levels of miRNAs that are typically found in biofluids, especially in unaffected controls. For RT-qPCR reactions, the relative measure of the concentration of a miRNA target is the quantification cycle (C_q_), the fractional cycle number at which a PCR amplification curve crosses a threshold line set within the exponential growth region of the amplification curve. If the amplification curve for an analyte never crosses the threshold line within 40–42 cycles of amplification, the analyte level is considered too low to be quantitated and the C_q_ is “undetermined.” C_q_ values are influenced by many factors, so they are not commutable values and cannot be directly compared between PCR reactions run under different conditions. Reporting of miRNA changes by relative quantitation of RT-qPCR data (normalizing C_q_ values to a control or reference sample C_q_) is not an optimal approach because baseline levels of many candidate tissue-selective miRNAs are very low or undetectable in biofluids and because there is no consensus set of small RNAs in serum or plasma that can be widely used for normalization [4]. Although not widely used in the published literature, absolute quantitation of miRNAs in biofluids is a documented method [5] that has application for reporting results in the exploratory stages of biomarker development. A common approach for measurement of miRNA biomarker candidates by absolute quantitation is the use of RT-qPCR to assay standard curves of synthetic RNA calibrators in parallel to samples for interpolation of unknowns. Analytical validation guidelines recommend the use of at least 6 non-zero calibrators per calibration curve [6]. Calibration curves should cover the dynamic range of the assay, which is a minimum of three orders of magnitude for PCR and ideally five to six [7]. miRNA can also be quantified using droplet digital PCR, an alternate PCR-based approach that doesn’t rely on standard curves [8, 9] and is just beginning to be applied to miRNA safety biomarker assessments.

**Fig. 1:**
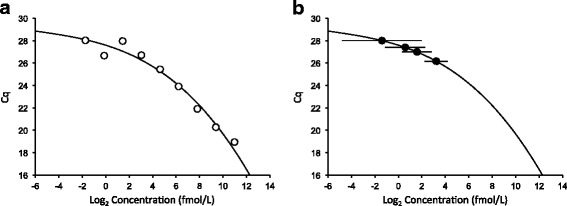
Examples of graphs aiding LLOQ determinations based on logistic modeling. **a** 3-parameter logistic model fit of calibration curve data. **b** Inverse predictions with 95% confidence intervals for sample data

The Health and Environmental Sciences Institute (HESI) Technical Committee on the Application of Genomics to Mechanism-Based Risk Assessment initiated a multi-site study to assess current practice for absolute quantification of miRNAs in biofluids using RT-qPCR [10]. Several cardiomyocyte-enriched miRNAs were measured in biofluids from a rat model of drug-induced cardiotoxicity by RT-qPCR using primarily TaqMan™ reagents. A three parameter logistic model was used to fit data from serial dilutions of calibrators (see Fig. 1a) for the estimation of copy numbers and 95% confidence intervals (CIs) in experimental samples (see Fig. 1b). A three parameter logistic (3PL) fit was determined to be the best approach for fitting all the observed types of calibration curve data (linear and non-linear) in this study to a single model. In the study, no-template controls (NTCs), which were analyzed in parallel to calibrators and study samples through all RT-qPCR steps, often had background C_q_ values instead of the “undetermined” calls that are expected for blank samples. The background signal in the NTC samples was consistent with the detection of low levels of amplified non-target sequence artifacts generated by RT-qPCR, such as primer-dimers or primer concatemers. The background signal in the NTC proved difficult to eliminate because it appeared to be influenced by multiple factors such as preamplification, reaction multiplicity, and reagent lots.

During the exploratory stage of miRNA biomarker development, guidelines are needed for determining assay thresholds that are appropriate for this method and purpose since circulating levels of tissue-selective miRNA can be at very low to potentially zero baseline levels. The lower limit of quantitation (LLOQ) is the lowest amount of an analyte in a sample that can be quantitatively determined with suitable precision and accuracy. Bioanalytical method validation approaches recommended for pharmacokinetic studies are a good starting point for biomarker measurements but are not necessarily fully applicable [6]. For bioanalytical assays, the LLOQ is typically determined based on the reagent blank response (e.g., at least five times higher) and a threshold for acceptable precision [6]. For miRNA measurements, approaches have been used that define an LLOQ based on the noise around the NTC values, in combination with the slope or linear region of a calibration curve. Kelnar et al. have defined a PCR-specific LLOQ as the NTC C_q_ value minus a factor (10 times the standard deviation of the NTC replicates divided by the negative slope of the standard curve) [11]. Hindson et al. defined an LLOQ as the lowest concentration tested that remained above or equal to both the lower limit of the linear range and the limit of detection (LOD) [8]. The linear range was determined by runs-testing and the LOD by the mean NTC C_q_ plus 2.479 times the standard deviation of NTC C_q_ values. Hughes et al. defined an LLOQ as the lowest calibration curve point with a mean C_q_ value less than the mean NTC C_q_ that, if included, does not cause the calibration curve to fall outside a range of 90–110% for PCR efficiency [12]. A simple approach recommended for use with Exiqon miRCURY LNA Universal RT-qPCR assays for miRNA defines the assay background as 5 C_q_ units below the NTC C_q_ or below 42 if the NTC signal is undetected [13].

In response to a current lack of consensus methods for LLOQ determinations that can be used for absolute quantitation of miRNA by RT-qPCR in investigational studies, we evaluated several different methods that are relatively simple and intuitive, and based on principles established for bioanalytical assays. Workflows for each approach were developed using a test set of 65 miR-1 calibration curves that included 10–12 serial dilutions and covered four orders of magnitude at 3-fold intervals (see Additional files 1, 2, 3, 4). This diverse set of calibration curves was generated at six sites following standard protocols that included variations in the degree of multiplexing and incorporated differences in linearity, slope, and detection of background signals in NTC samples. LLOQ determinations were limited to measured points and were calculated on a per-run basis to match projected future workflows. The two workflows that were developed are described further below.

## Results

### LLOQ determination based on logistic modeling

A logistic model is useful for analyzing calibration data, as it finds a smooth underlying S-shaped curve to the data along with estimates of the noise around it. It furthermore enables inverse prediction, in which an x-value is determined from an observed y-value by reflecting off of the fitted curve. When using the logistic modeling approach for LLOQ determinations, it is advisable to construct and study various graphs in order to ensure the method is performing as intended as well as to discover any unusual data patterns and to tune threshold parameters. Figure 1 provides two such example graphs. Figure 1a plots a 3-parameter logistic fit to data for one of the runs; this is a case where the curve fits the data fairly well. Figure 1b is from the same data as 1a, and illustrates the degree of uncertainty inherent in model-based inverse prediction by means of arrows representing 95% confidence intervals on each inverse-predicted point. Inverse prediction makes LLOQ determination relatively straightforward after determining a suitable measure and threshold for error. We investigated two ways to do this. The first computes the percent relative error (*δ*) of inverse predictions (C_p_) of known concentrations (C_k_), using the following equation and a threshold of 20%.

1 $$ \delta =100\;\left|\frac{C_k-{C}_p}{C_k}\right| $$

**Fig. 2:**
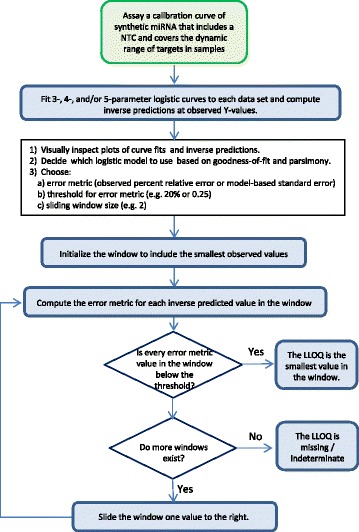
Decision tree workflow for LLOQ determinations based on logistic modeling

The second option uses the model-based estimate of the standard error of the inverse prediction along with a threshold of 0.25. The standard error is computed using a first-order Taylor-series expansion of the inverse model eq. [14]. In both cases we look for two consecutive values that are below the threshold and choose the LLOQ to be the smallest observed concentration among all such pairs or set it to missing if no such value exists. The respective thresholds of 20% and 0.25 seem to be reasonable defaults and can be adjusted depending upon the desired application of the LLOQ values. If the logistic model does not fit the data well, e.g., in cases of poor data quality, the LLOQ will be indeterminate using the logistic model. Figure 2 depicts a workflow for the logistic model method with its two options.

### LLOQ determination based on baseline noise

Quantitation limits for analytical assays have also been based on signal-to-noise ratio [15]. A signal-to-noise ratio of 10 is a typical constant used to define the minimum concentration at which an analyte can be reliably quantified. We tested one approach that is based on defining an LLOQ as the nearest measured point that is ten-fold higher than the baseline noise level, with baseline noise being defined as the signal in the NTC sample in RT-qPCR assays. If there is no measurable background noise in the samples (i.e., C_q_ values for the NTC sample are assigned an “undetermined” call by the qPCR instrument software), the baseline signal could alternately be defined by the lowest detectable signal determined by the calibrators.

**Fig. 3:**
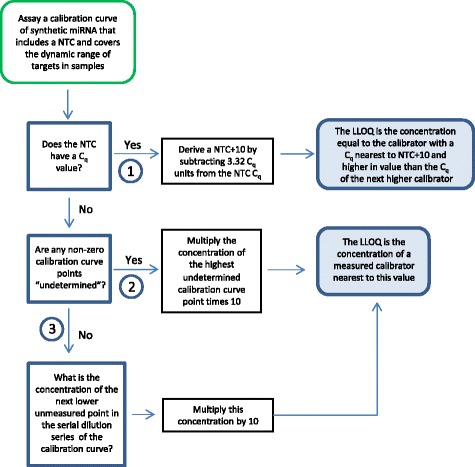
Decision tree workflow for LLOQ determinations based on baseline noise

**Fig. 4:**
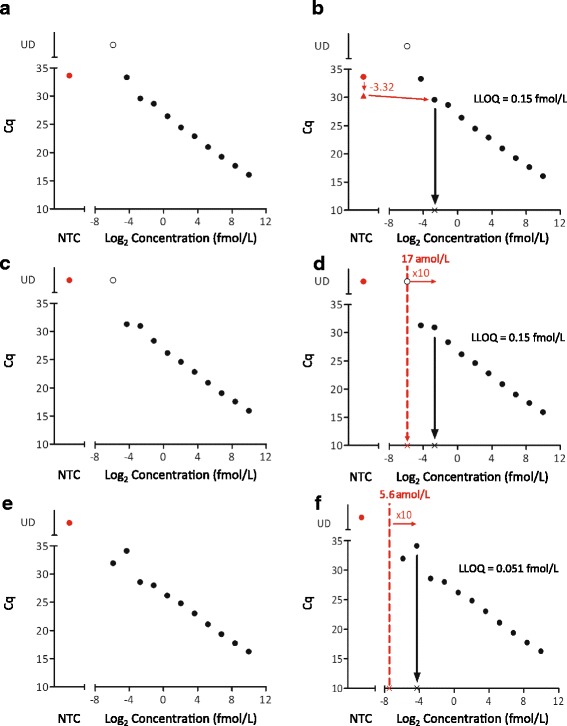
Examples of LLOQ determinations made using the baseline noise decision tree. The data is from the 3 replicates of miR-1 calibration curves run at one site on different days that covers the 3 options in the workflow. (**a** + **b**) Example of data that meets option 1: the NTC has a C_q_ value from which 3.32 is subtracted to derive a NTC + 10. The calibrator dilution with a C_q_ nearest the NTC + 10 is the LLOQ. (**c** + **d**) Example of data that meets option 2: the C_q_ for the lowest calibration curve point is undetermined. The lowest calibrator concentration is multiplied by 10 and the nearest calibrator concentration is the LLOQ. (**e** + **f**) Example of data using option 3. The concentration of the next unmeasured serial dilution point is calculated and multiplied by 10. The nearest measured calibrator concentration is the LLOQ. Black circles: calibration curve points. Red circles: NTC values. Red triangle: NTC + 10

A workflow developed for LLOQ determinations based on baseline noise includes three decision nodes that cover the types of data found in the test set (Fig. 3). The first decision node asks if a C_q_ value is determined for the NTC. If yes, a factor of 10 (3.32 C_q_ units assuming a doubling of product per cycle) is subtracted from the mean NTC C_q_ to derive the NTC + 10. The concentration of the lowest measured point in the calibration curve with a mean C_q_ less than the NTC + 10 but higher in value than the C_q_ of the next higher calibrator is defined as the LLOQ. The second decision node asks if a mean C_q_ is “undetermined” for both the NTC sample and for at least one of the calibration curve dilutions. In that case, the highest calibration curve point with an “undetermined” C_q_ value is defined as the baseline noise level. The concentration of that point is multiplied by a factor of ten and the LLOQ is defined as the nearest measured point to this value. If the NTC C_q_ has an “undetermined” value but C_q_ values are assigned to all calibration curve points, the next lower dilution of the calibration curve (if it had been measured) is assumed to be the limit for signal-response measurements. For this situation, the concentration of the next dilution point in the series is multiplied by a factor of ten and the LLOQ is defined as the measured point nearest to this value. Examples of LLOQs determined using data that applies to the three decision nodes are given in Fig. 4. The baseline noise approach can be used to set thresholds for inverse predictions estimated from logistic modeling or log-linear regression of calibration curve data.

### Comparison of LLOQ values determined using two workflows with a multi-site calibration curve dataset

**Fig. 5:**
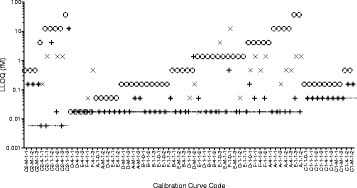
LLOQ determinations from two workflows for a multi-site set of miR-1 calibration curves. The lowest calibrator concentration in each standard curve (5.7 amol/L, 16.9 amol/L, or 50.8 amol/L) is indicated by the dashed line. LLOQs were calculated by the baseline noise workflow (O) or the logistic model workflow using the relative error option (+) or the standard error option (X). The calibration curve code conveys the presence of differences in site, assay multiplicity, and round among the dataset

Figure 5 illustrates the differences between the two workflows by plotting LLOQ values determined for the 65 miR-1 calibration curves in the test set. These values are also available in Additional file 5. The baseline noise workflow was the more conservative of two workflows. Most (92%) of the 65 LLOQs calculated by the baseline noise workflow were higher than the lowest calibrator concentration. Decision node 1 of the baseline noise workflow applied to the majority (69%) of the test set calibration curves that had C_q_ values were reported for the NTCs. For the logistic model-based approach, the lowest calibrator concentration was the same as the LLOQ for the majority of calculations using the relative error option (72%) or standard error option (54%) for this dataset. The two different options within the logistic model workflow produced the same result for 43 of the 65 calibration curves in the test set. When the results differed, the LLOQs determined by the standard error method were higher in magnitude for 21 of the 22 examples than the LLOQs determined by the relative error method, which was the least conservative of the approaches tested.

### Application of LLOQ workflows for setting thresholds on miRNA values in an RT-qPCR absolute quantitation data set

**Fig. 6:**
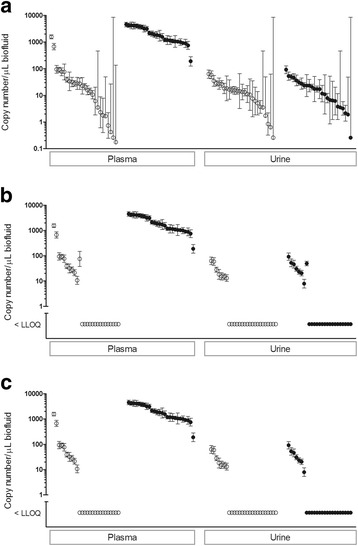
Inverse predictions from 5 sites for miR-208a-3p levels with 95% confidence intervals in biofluids from pooled control (open circles) and 24 h isoproterenol (closed circles) treatment groups. In (**a**), no LLOQ threshold was applied. In (**b**), LLOQ thresholds were applied based on logistic model standard error and on baseline noise in (**c**). In total, 50/104 measurements were above the LLOQ for the baseline noise model and 52/104 for both of the logistic models. The methods agree on the common 50 and the two logistic methods are in exact agreement.

The two LLOQ workflows were applied to a use case consisting of an independent inter-laboratory study described by Thompson et al. [10]. The dataset consists of 14 miRNA calibration curves and 104 unknowns generated at 5 sites by absolute quantitation of cardiac-enriched miR-208a-3p in plasma and urine samples from control and isoproterenol-treated rats. This dataset is suitable for comparing LLOQ workflows because baseline levels of miR-208a-3p in plasma have been observed to be near detection limits in several studies of its performance as a preclinical and clinical biomarker of cardiac injury [16, 17]. MiR-208a-3p was assayed in four biofluid samples on three separate days and quantitated from calibration curves run in parallel. Each calibration curve consisted of eight or ten 3-fold serial dilutions of a common stock of 2 pmol/L synthetic miR-208a-3p RNA, with the lowest dilution set at 0.3 fmol/L for 4 sites and at 0.03 fmol/L for one site (see Additional file 6). These lowest calibrator points are equivalent to 36.7 and 4.1 copies per μL biofluid, respectively. A 3-parameter logistic model was used to estimate copy numbers of miR-208a per μL and 95% CIs (Fig. 6a). Both of the LLOQ workflows used the calibration curve data and/or NTC values from the study to determine an LLOQ for each run. The LLOQs were used to set a lower threshold for miR-208a levels predicted from each run. miR-208a-3p values that were below the LLOQs determined using the logistic model with the standard error option or the baseline noise approach are graphed below the y-axis in Fig. 6b and c, respectively. The results for the relative error option were identical to the standard error option for this dataset and are not shown. Many of the miR-208a-3p measurements in control plasma or in urine were determined to be below LLOQs determined by either approach. For this dataset, nearly all (13/14) of the LLOQs determined using the standard error option, all 14 of the LLOQs determined using the relative error option, and 5/14 of the LLOQ determined using the baseline noise workflow were the same as the lowest calibrator concentration (see Additional file 7). Within the logistic model workflow, the standard error option appeared to be more conservative than the relative error option for this dataset using the aforementioned 0.25 and 20% thresholds, respectively.

## Discussion

As part of a study on inter-laboratory variance in the absolute quantitation of miRNAs in biofluids using RT-qPCR, it became evident that there was a lack of clarity on how to establish a LLOQ for this type of data. It is useful to identify approaches that can be retrospectively applied to the types of datasets that were generated for this multi-site study. For example, in the standard protocol for the multi-site study, technical replicates were run at the PCR step but not at the RT step. LLOQ approaches that are based on error around the NTC C_q_ values would not be applicable to these datasets because both RT and PCR steps need to be included in the error estimate. The two workflows described in this report were developed using principles established for bioanalytical assays. The approaches are based on the logistic modelling of calibration curve data or the NTC C_q_ values for LLOQ determinations. When applied to a large dataset of calibration curves that were designed to exceed the linear range of the RT-qPCR assays, the two workflows demonstrated general utility, albeit with some differences in conservatism for calculating a LLOQ. These approaches for LLOQ determinations should be applicable to other types of nucleic acid measurements that use calibration curves for quantitation including injury-related miRNAs at sites other than heart and miRNA within tissues and cell lines.

Levels of cardiac-selective miR-208a-3p were measured in biofluids as part of a multi-site study with the expectation based on published literature [16, 17] that low levels would be observed in control plasma and increased levels observed in plasma within 4–24 h of administration of a subcutaneous dose of isoproterenol that induces moderate cardiac injury in male Wistar rats. The levels of miR-208a-3p in the urine of control rats or rats with isoproterenol-induced cardiac injury were an unknown. In this study, we observed that control plasma levels of miR-208a-3p were relatively low and varied by 1000-fold among sites and/or runs, and that the lowest levels (<10 copies/μL biofluid) were associated with large error estimates predicted by the 3-parameter logistic model. When LLOQ thresholds were applied to this dataset that were based on the logistic standard error workflow, it follows that the miR-208a-3p predicted values for unknowns with large confidence intervals in control plasma and in urine are below the LLOQ. The baseline noise workflow for LLOQ determinations that sets a threshold at an interval below the NTC value has a similar effect in filtering out the lower miR-208a-3p estimates with high error estimates. Although there is a rationale for retaining values below the LLOQ to report estimates instead of no results [18], this use case illustrates why error estimates should be calculated and provided for inverse predictions of miRNA copy number concentrations for exploratory biomarker studies.

## Conclusions

We developed and tested  two workflows that provide guides for LLOQ determinations and fill a gap in methods that are appropriate for exploratory use of miRNAs as safety biomarkers in drug development. These two workflows are summarized in Additional file 8. We include sample data sets (Additional files 1, 2, 3, 4 and 6) and results tables (Additional files 5 and 7) to allow investigators to train themselves in the use of these guides.

## Methods

### Synthetic microRNA standards used in the test set

Concentrated stocks of rno-miR-1-3p, rno-miR-16-5p, rno-miR-208a-3p, and rno-miR-499-5p RNA were synthesized, HPLC-purified, quantified, and mixed by a contracting lab (IDT, Coralville, IA), and distributed to test sites as a pooled stock solution comprised of equimolar amounts of four miRNAs. A dilution series was prepared by ten 3-fold serial dilutions of a 1 pmol/L stock of the equimolar pool to span an input range of 1 pmol/L to 17 amol/L. The calibration curve dilutions were prepared in water with carrier non-mammalian RNA (MS bacteriophage RNA, Roche Diagnostics) at a concentration of 0.5 ng/μL.

### RT-qPCR protocol for the test set

The test set protocol includes three arms that involve three different degrees of primer pool multiplicity in the reverse transcription and preamplification steps (singleplex, 4-plex, and Megaplex). A single RT reaction was run per arm for 12 samples: an 11-point serial dilution curve of synthetic rno-miR-1-3p and a no template control. The RT reactions were preamplified in replicate on 3 separate days, followed by qPCR. The singleplex assay contained TaqMan microRNA assay reagents for rno-mir-1 (Assay No. 002064). Each 15 μL RT reaction included 0.15 μL 100 mmol/L dNTPs, 1.5 μL 10X RT Buffer, 0.19 μL RNase inhibitor (20 U/μL), 6.16 μL H_2_O, 3 μL 5× RT primer, 1 μL Multiscribe reverse transcriptase, and 3 μL calibrator RNA or water, and reactions were run in a thermal cycler at 16 °C, 30 min; 42 °C, 30 min; 85 °C, 5 min; 4 °C, hold. The singleplex RT reaction was either preamplified and analyzed by qPCR or analyzed directly by qPCR. Preamplification reactions had 2.5 μL RT reaction, 12.5 μL 2× TaqMan Pre-AMP Master Mix, 6.25 μL 0.2× miR-1 TaqMan assay, and 3.75 μL H_2_O in a total volume of 25 μL. The reactions were run in a thermal cycler for 14 cycles of [95 °C, 15 s; 60 °C, 4 min] and then placed immediately on ice. The preamplification reaction products were diluted 1:20 in 0.1× TE and 5 μL of diluted preamplification product, undiluted RT reaction, or 1:200 diluted RT reaction was added to a 20 μL qPCR reaction containing 1 uL 20× TaqMan miR-1 microRNA assay, 10 μL 2× Universal Master Mix II no UNG, and 4 μL H_2_O. Reactions were run at 95 °C for 10 min, followed by 40 cycles of [95 °C, 15 s; 60 °C, 60 s] in an Applied Biosystems 7900HT or ViiA7 qPCR instrument. The same qPCR run parameters were used for the singleplex, 4-plex, and Megaplex qPCR reactions.

The 4-plex RT reaction used pooled RT primers from rno-miR-1-3p, rno-miR-208a-3p, rno-miR-499-5p, and rno-miR-192-5p TaqMan microRNA assays. The 15 μL 4-plex RT reactions consisted of 0.15 μL 100 mmol/L dNTPs, 1.5 μL 10× RT Buffer, 0.19 μL RNase inhibitor (20 U/μL), 3.16 μL H_2_O, 6 μL 1.25× pooled RT primer, 1 μL Multiscribe reverse transcriptase, and 3 μL calibrator RNA, and were run in a thermal cycler at 16 °C, 30 min; 42 °C, 30 min; 85 °C, 5 min; 4 °C, hold. The 4-plex RT reaction products (2.5 μL) were added to a 25 μL preamplification reaction containing 12.5 μL 2× TaqMan Pre-AMP Master Mix, 6.25 μL 0.2× pooled TaqMan assays for rno-miR-1-3p, rno-miR-208a-3p, rno-miR-499-5p, and rno-miR-192-5p, and 3.75 μL H_2_O in a total volume of 25 μL. The reactions were run in a thermal cycler for 14 cycles of [95 °C, 15 s; 60 °C, 4 min] and placed immediately on ice. The 4-plex preamplification products were diluted 1:20 and 5 μL was combined with 1 uL 20× TaqMan miR-1 microRNA assay, 10 μL 2× Universal Master Mix II no UNG, and 4 μL H_2_O.

The Megaplex RT reactions combined 0.8 μL 10× Megaplex RT primers for Rodent Pool A (Catalog No 4399970), 0.2 μL 100 mmol/L dNTPs, 1.5 μL Multiscribe reverse transcriptase, 0.8 μL 10X RT Buffer, 0.9 μL 25 mmol/L MgCl_2,_ 0.1 μL RNase inhibitor (20 U/μL), 0.2 μL H_2_O, and 3 μL calibrator RNA. The RT reactions were incubated on ice for 5 min and run in a thermal cycler with a heated lid for 40 cycles of [16 °C, 2 min; 42 °C, 1 min; 50 °C, 1 s] followed by 85 °C, 5 min and held at 4 °C. Each Megaplex preamplification reaction contained 12.5 μL 2× TaqMan Pre-AMP Master Mix, 2.5 μL 10× Megaplex PreAMP primers for Rodent Pool A (Catalog No 4399203), 7.5 μL H_2_O, and 2.5 μL Megaplex RT reaction product. The Megaplex preamplification reactions were run in a thermal cycler at 95 °C, 10 min; 55 °C, 2 min; 72 °C, 2 min, followed by 12 cycles at [95 °C, 15 s; 60 °C, 4 min], followed by 99.9 °C, 10 min and held at 4 °C. Each 25 μL reaction was diluted by adding 75 μL 0.1× TE pH 8.0. The Megaplex preamplification products were further diluted 1:20 and 5 μL was assayed by qPCR by adding 1 μL 20X TaqMan miR-1 microRNA assay, 10 μL 2× Universal Master Mix II no UNG, and 4 μL H_2_O.

### Site differences in the test set

Six sites participated in the study used for the test set. All six ran the singleplex and 4-plex arms and four sites also ran the Megaplex arm. Three different brands of thermal cycler were used by the six sites for the RT and preamplification steps. Three sites used the Applied Biosystems 7900HT system and three sites used the Applied Biosystems ViiA7 system for qPCR steps. The one reported variable within a site was the use of three different lots of TaqMan PreAmp MasterMix by Site C.

### Software

Calculations were performed in Excel and JMP, and graphics were created in Excel, GraphPad, and JMP. A free JMP add-in called “Calibration Curves” is available at https://community.jmp.com/docs/DOC-6285 to aid in fitting 3PL curves and performing inverse prediction.

## Additional files

Additional file 1: Multi-site miRNA calibration curve dataset for the singleplex no preamp RT-qPCR pipeline. Results for the miR-1 calibration curves that were generated at six sites (A-F) as described in Methods are provided. (CSV 1 kb)

Additional file 2: Multi-site miRNA calibration curve dataset for the singleplex preamp RT-qPCR pipeline. (CSV 2 kb)

Additional file 3: Multi-site miRNA calibration curve dataset for the 4-plex preamp RT-qPCR pipeline (CSV 2 kb)

Additional file 4: Multi-site miRNA calibration curve dataset for the Megaplex preamp RT-qPCR pipeline. (CSV 1 kb)

Additional file 5: LLOQ determinations based on Logistic Modeling and Baseline Noise approaches for datasets in Additional files 1-4 and plotted in Fig. 5. (CSV 2 kb)

Additional file 6: miR-208a-3p calibration curve dataset from 5 sites used for LLOQ determinations in Additional file 5. (CSV 11 kb)

Additional file 7: Predicted miR-208a-3p values in plasma and urine from control and isoproterenol-treated rats and the corresponding LLOQs determined using two workflows and plotted in Fig. 6. (CSV 10 kb)

Additional file 8: Summary of Logistic Modeling and Baseline Noise approaches to LLOQ determinations. (PDF 55.1 KB)
