# Supplementary material for: Two approaches for estimating the lower limit of quantitation (LLOQ) of microRNA levels assayed as exploratory biomarkers by RT-qPCR
Source: BMC Biotechnol. 2018 Feb 2;18:6. doi: 10.1186/s12896-018-0415-4 (PMC5796571; doi:10.1186/s12896-018-0415-4)
Supplement: Supplementary file 8 — Summary of Logistic Modeling and Baseline Noise approaches to LLOQ determinations. (PDF 55.1 KB) [file 12896_2018_415_MOESM8_ESM.pdf]

**Approach**	**Logistic Modeling**	**Baseline Noise**
**Overview**	Fits a logistic model to calibration curves and uses resulting estimates to determine LLOQ.	Uses No Template Control (NTC) values and a set of well-proven empirical rules to determine LLOQ.
**Caveats and Concerns**	Choosing a specific logistic model, error metric, threshold, and sliding window requires some consideration. The model may not fit well to poor-quality data.	NTC samples should be assayed appropriately and rules checked and followed closely to ensure good results.
